# Smyd3-PARP16 axis accelerates unfolded protein response and vascular aging

**DOI:** 10.18632/aging.103895

**Published:** 2020-11-03

**Authors:** Di Yang, Qing Wang, Gang Wei, Jiaxue Wu, Yi Chun Zhu, Qing Zhu, Ting Ni, Xinhua Liu, Yi Zhun Zhu

**Affiliations:** 1 Department of Pharmacology, Human Phenome Institute, School of Pharmacy, Fudan University, Shanghai, P.R. China; 2 State Key Laboratory of Quality Research in Chinese Medicine and School of Pharmacy, Macau University of Science and Technology, Macau, P.R. China; 3 Shanghai Key Laboratory of Bioactive Small Molecules and Research Center on Aging and Medicine, Department of Physiology and Pathophysiology, School of Basic Medical Sciences, Fudan University, Shanghai, P.R. China; 4 State Key Laboratory of Genetic Engineering and MOE Key Laboratory of Contemporary Anthropology, Collaborative Innovation Center of Genetics and Development, Human Phenome Institute, School of Life Sciences and Huashan Hospital, Fudan University, Shanghai, P.R. China; 5 State Key Laboratory of Genetic Engineering, School of Life Sciences, Fudan University, Shanghai, P.R. China; 6 School of Pharmacy, Nantong University, Nantong, P.R. China

**Keywords:** vascular endothelial cell, PARP16, angiotensin II, endoplasmic reticulum, Smyd3

## Abstract

Vascular endothelial cell senescence and endoplasmic reticulum (ER) stress induced unfolded protein response (UPR) are two critical contributors to individual aging. However, whether these two biological events have crosstalk and are controlled by shared upstream regulators are largely unknown. Here, we found PARP16, a member of the Poly (ADP-ribose) polymerases family that tail-anchored ER transmembrane, was upregulated in angiotensin II (Ang II)-induced vascular aging and promoted UPR. Further, PARP16 was epigenetically upregulated by Smyd3, a histone H3 lysine 4 methyltransferase that bound to the promotor region of *Parp16* gene and increased H3K4me3 level to activate its host gene’s transcription. Intervention of either Smyd3 or PARP16 ameliorated vascular aging associated phenotypes in both cell and mice models. This study identified Smyd3-PARP16 as a novel signal axis in regulating UPR and endothelial senescence, and targeting this axis has implications in preventing vascular aging and related diseases.

## INTRODUCTION

Cellular senescence and unbalance of protein homeostasis, referred to as proteostasis, are two important hallmarks of aging and the key risk factor for age-related diseases such as cardiovascular disorder and cancer [1–3]. Subsequent studies identified that proteostasis contributes to the initiation of aging [4, 5]. Thus, possible therapeutic interventions should be conducted to improve proteostasis and prevent pathological aging.

Endoplasmic reticulum (ER) is a central organelle involved in protein folding, maturation and quality control [6]. In non-stress conditions, the ER chaperone, Bip binds to the luminal domains of three ER transmembrane sensors: inositol-requiring enzyme 1 (IRE1), protein kinase R [PKR]-like ER kinase (PERK), and activating transcription factor 6 (ATF6) to keep them inactive [7]. Many genetic, environmental, or aging-related insults alter the ER homeostatic balance, leading to ER stress. To cope with ER stress, accumulated protein in the ER lumen causes dissociation of Bip from these sensors which activate an integrated signaling network known as the unfolded protein responses (UPR). Activation of UPR initiates 3 distinct UPR branches to re-establish homeostasis of protein folding in different physiological and pathological conditions [8] [9]. Importantly, the occurrence of abnormal levels of ER stress has been reported in vascular dysfunction. However, the complex interplay between ER stress and vascular aging is far from being elucidated. A better understanding of the molecular and cellular mechanisms underlying ER stress and vascular aging will provide a list of candidate molecules which could be considered as targets for specific interventions aimed at delaying vascular aging-related diseases [10].

Vascular endothelial cell senescence is regarded as a response to endothelial cell dysfunction and contributes to the vascular diseases [11]. Extensive studies have demonstrated that ER stress occurs in the pathogenesis of various aging-related cardiovascular diseases such as atherosclerosis, hypertension and ischemic heart disease [12]. Since vascular endothelial cell senescence and loss of proteostasis mediated by ER stress are critical contributors of individual aging and aging-related cardiovascular diseases [8], the question arises of whether these two biological events have crosstalk and are controlled by shared upstream regulators. To address their relationship, we search the upstream genetic and/or epigenetic regulator in vascular cellular senescence, which was reported to be one of the most important contributors to individual aging and age-related cardiovascular diseases such as atherosclerosis and hypertension [13].

PARP16 (also known as ARTD15), a member of the PARP/ARTD family, is a tail-anchored protein located at the endoplasmic reticulum membrane [14], and required for activation of the functionally related ER stress sensors PERK and IRE1 during the UPR [15]. Besides, the epigenetic mechanism is reported to modulate both longevity and mitochondrial proteostasis throughout life. Our previous study found that Smyd3, a lysine methyltransferase, induced vascular cell senescence-associated phenotypes by directly binding to promotor region of p21 coding gene *Cdkn1a* and leading to increased H3K4me3 level and gene expression. However, it remains unclear whether Smyd3 regulates *Parp16* gene transcription, which affects ER stress and endothelial senescence.

Here, we showed that PARP16 was upregulated and played a key role in promoting UPR and endothelial cell senescence. More importantly, PARP16 was epigenetically upregulated by Smyd3 and intervention of either Smyd3 or PARP16 could ameliorate vascular aging associated phenotypes in both cell and mice models. This study identified Smyd3-PARP16 as a novel signal axis in regulating UPR and endothelial senescence, and targeting this axis has implications in preventing vascular aging and related diseases.

## RESULTS

### PARP16 is involved in Ang II-induced endoplasmic reticulum (ER) stress and vascular senescence

**Figure 1:**
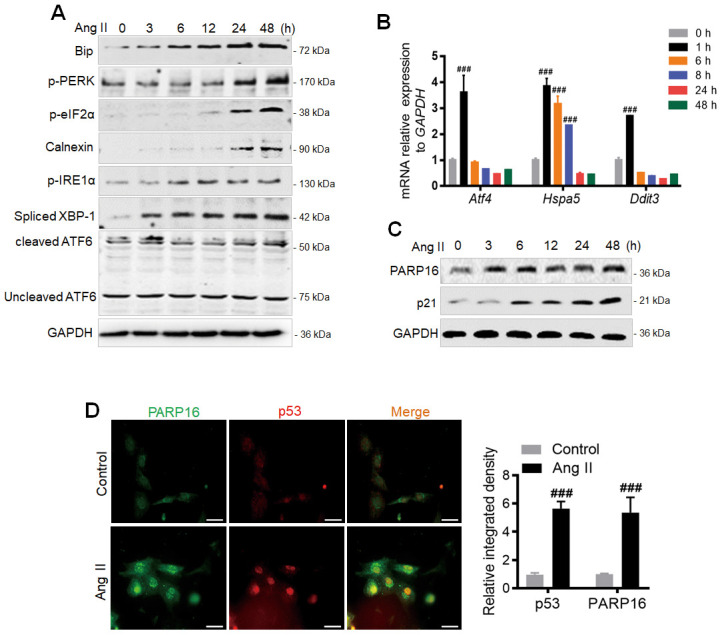
**PARP16 is involved in Ang II-induced endoplasmic reticulum (ER) and endothelial cells senescence.** (**A**) ER markers were upregulated after Ang II stimulation in RAECs. At 0, 3, 6, 12, 24 and 48 h after Ang II (2 μM) administration, cells extracts were collected for determining the protein levels. Data shown are representative of data from at least three different replicates. (**B**) qRT-PCR analysis of the UPR target genes (*Hspa5*, *Ddit3*, *Atf4*) after Ang II stimulation for indicated time in RAECs. Data shown are technically representative of data from at least three different replicates; ^###^*p* < 0.001 *vs.* 0 h. (**C**) PARP16 and senescence-associated marker p21 were upregulated after Ang II stimulation in RAECs. At 0, 3, 6, 12, 24 and 48 h after Ang II (2 μM) administration, cells extracts were collected for determining the protein levels. Data shown are representative of data from at least three different replicates. (**D**) Immunofluorescence double staining of PARP16 and p53, and quantitative analysis in RAECs upon Ang II treatment, ^###^*p* < 0.001 *vs.* control. ‘DAPI’ represents DAPI staining of nuclei (DNA) throughout the manuscript.

Angiotensin II (Ang II), a major hormone that mediates atherosclerosis and hypertension [16], not only promotes cellular senescence [11], but also induces ER stress. Based on the circulating levels of Ang II detected in patients with hypertension and significantly increased concentration of Ang II in pathological state, we selected Ang II (2 μM) to induce Rat Aorta Endothelial Cells (RAECs) as a vascular aging cell model. We found multiple ER stress markers including Bip, p-PERK, p-eIF2α, p-IRE1α, cleaved ATF6, Calnexin and Spliced XBP-1 were increased in time-dependent manner (Figure 1A) [17, 18]. Furthermore, the UPR target genes *Atf4* (encoding ATF4 protein) and Hspa5 (encoding Bip protein) were significant upregulated while the activation of apoptotic gene *Ddit3* (encoding CHOP protein) was modestly induced after Ang II treatment for 1 h (Figure 1B). We next examined which factor could be the upstream regulator mediating ER stress and cellular senescence during Ang II induction. PARP16 is a tail-anchored ER transmembrane protein required for activation of the functionally related ER stress [19]. Interestingly, PARP16 showed a continuous increase of protein level during Ang II induced RAEC senescence (Figure 1C), suggesting its potential role in regulating senescence through UPR of ER. Immunofluorescence double staining showed that the co-expression of PARP16 and p53 induced by Ang II appeared in RAECs (Figure 1D). To confirm that upregulation of PARP16 also exists in other senescence vascular cells, we selected Ang II induced Rat Vascular Smooth Muscle Cells (VSMCs) since senescent VSMCs also promote the pathological process of vascular diseases [20, 21]. Importantly, PARP16 also displayed a progressive elevation after induction of Ang II in VSMCs, together with increased levels of vascular senescence markers (p21 and p16), senescence-associated secretory phenotypes (SASP) (VCAM-1, COX-2 and IL-6) and ER stress markers (p-PERK, Bip, p-eIF2α, p-IRE1α, Spliced XBP-1, cleaved ATF6 and Calnexin) (Supplementary Figure 1A). Consistent with this, the UPR target genes (*Hspa5, Ddit3, Atf4*) were also increased in Ang II induced VSMCs (Supplementary Figure 1B). These results support that PARP16 upregulation may contribute to the vascular senescence.

### PARP16 overexpression promotes RAECs senescence and endoplasmic reticulum stress

**Figure 2:**
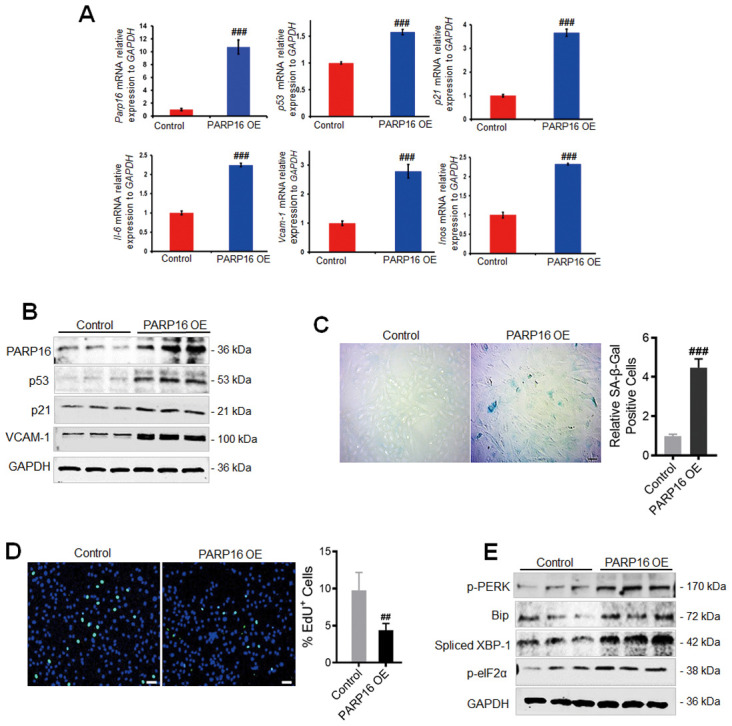
**PARP16 overexpression promotes RAECs senescence and endoplasmic reticulum.** RAECs were transfected with lentivirus-mediated PARP16 cDNA (PARP16 OE) for 72 h, *Parp16*, *p21*, *p53*, *Il-6*, *Vcam-1*, *Inos* mRNA level were confirmed by qRT-PCR. Data shown are representative of data from at least three different replicates; ^###^*p* < 0.001 *vs.* control. (**A**); cell lysates were immunoblotted with antibody against PARP16, p53, p21 and VCAM-1. GAPDH serves as internal control (**B**); SA-β-Gal staining (**C**) and EdU incorporation assay (**D**) of RAECs upon overexpression of PARP16, ^##^*p* < 0.01, ^###^*p* < 0.001 *vs.* control, n=5; (**E**) p-PERK, p-eIF2α, Bip and Spliced XBP-1 level were determined in PARP16 overexpressing cells. Data shown are representative of data from at least three different replicates.

To examine whether PARP16 plays a causal role in regulating ER stress and vascular cell senescence, we overexpressed PARP16 in RAEC cells (Figure 2A). Intriguingly, overexpression of PARP16 in RAECs led to elevated mRNA and protein levels of senescence markers p53, p21, and VCAM-1, but also increased mRNA levels of senescence-associated secretory phenotype genes including *Il-6* and *Inos genes* (Figure 2A, 2B). More percent of cells displayed SA-β-Gal staining when PARP16 overexpressed (Figure 2C). Decreased EdU incorporation level was also observed in PARP16 overexpressing cells (Figure 2D). The above evidence supports that higher level of PARP16 alone promotes multiple key phenotypes that associated with vascular cell senescence. To further determine whether PARP16 functions during UPR, we overexpressed PARP16 in RAECs and examined the key markers of ER stress induced UPR. Consistent with the unfolded protein response upon Ang II induction, overexpression of PARP16 led to increased levels of p-PERK, Spliced XBP-1, p-eIF2α and Bip, the well-known ER stress markers (Figure 2E). These results indicate that PARP16 can promote both unfolded protein response and cellular senescence.

### Knocking down or inhibiting of PARP16 mitigates Ang II-induced RAECs senescence and ER stress

**Figure 3:**
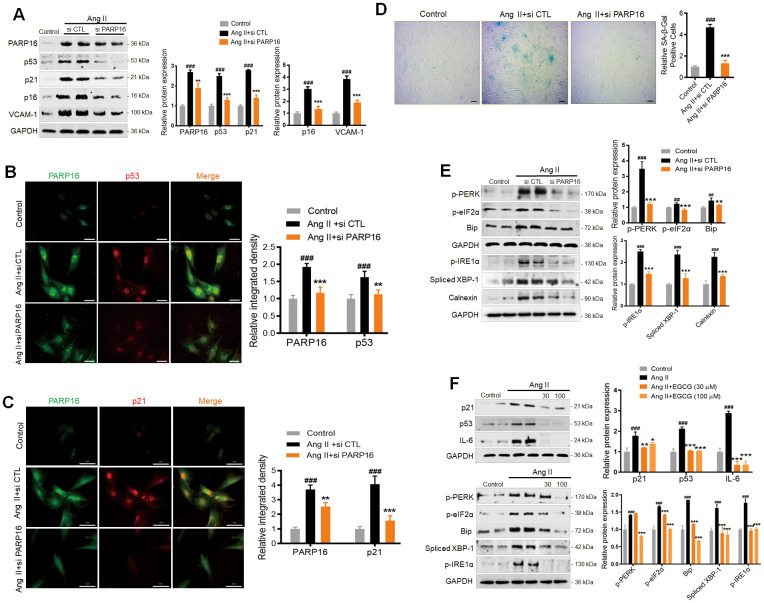
**Knocking down or inhibition of PARP16 mitigates Ang II-induced RAECs senescence and endoplasmic reticulum (ER) stress.** (**A**–**E**) PARP16 knockdown reversed Ang II-induced RAECs senescence and endoplasmic reticulum stress. Senescence-associated markers (VCAM-1, p16, p21, and p53) were assayed by Western blot for RAEC cells transfected with control (si CTL) or PARP16 siRNA before and after Ang II (2 μM) treatment for 48 h (**A**); Immunofluorescence double staining of PARP16 and p53 (**B**), and Immunofluorescence double staining of PARP16 and p21 (**C**); SA-β-Gal staining for RAEC cells (**D**); ER-associated markers (Bip, p-PERK, p-eIF2α, p-IRE1α, spliced XBP-1 and Calnexin) were assayed by Western blot for RAEC cells transfected with control or PARP16 siRNA before and after Ang II treatment (**E**). (**F**) PARP16 inhibitor EGCG reversed Ang II-induced RAECs senescence and endoplasmic reticulum stress. p21, p53, IL-6, Bip, p-PERK, spliced XBP-1, p-IRE1α and p-eIF2α were assayed by Western blot for Ang II-induced RAEC cells with or without PARP16 inhibitor (EGCG) at different concentration. GAPDH serves as internal control. All data were shown as mean ± S.D of at least 4 independent experiments. ^##^*p* < 0.01, ^###^*p* < 0.001 *vs.* control; ^*^p < 0.05, ^**^p < 0.01, ^***^p < 0.001, *vs.* Ang II+si CTL or Ang II treated cells.

To further connect our findings with classic UPR branches, we used the pharmacological inhibitors of the following UPR branches: a specific IRE1 inhibitor 4μ8C and PERK inhibitor ISRIB, which reverse effects of eIF2α phosphorylation. As shown in Supplementary Figure 2, both 4μ8C and ISRIB could inhibit the Ang II-induced upregulation of senescence marker (p53, p21) and SASP (VCAM-1), and decrease the key markers of ER stress, suggesting that either IRE1 inhibition or reversing effects of eIF2α phosphorylation prevented Ang II-induced RAECs senescence. Since PARP16 can promote both UPR and cellular senescence, we next asked whether blocking PARP16 could mitigate Ang II-induced UPR and senescence phenotypes. Excitingly, knocking down of PARP16 in RAECs by two different siRNAs could block the Ang II-induced increase in senescence-associated molecular phenotypes including p53, p21, p16, and VCAM-1 (Figure 3A). Immunofluorescence double staining showed that the co-expression of PARP16 and senescence markers (p53 and p21) induced by Ang II disappeared upon knockdown of PARP16 (Figure 3B, 3C). In addition, lower PARP16 expression led to reduced SA-β-Gal activity upon Ang II induction (Figure 3D). In addition to its causal role in regulating senescence phenotypes, knocking down of PARP16 also blocked ER stress related molecular phenotypes including Bip, p-PERK, p-eIF2α, p-IRE1α, Spliced XBP-1 and Calnexin in Ang II-treated RAECs (Figure 3E). In addition to RNA interference, enzymatic inhibition was also applied to demonstrate the regulation function of PARP16. Epigallocatechin gallate (EGCG) has been reported is a potential inhibitor of PARP16, which suppressed the ER stress-induced phosphorylation of PERK and the transcription of UPR-related genes [22]. To explore whether EGCG may be a potential strategy for preventing vascular aging, EGCG was added into Ang II-induced RAECs and the results showed that inhibition of PARP16 reversed senescence-related phenotypes (p53, p21 and IL-6) and ER stress markers (Bip, p-PERK, p-eIF2α, p-IRE1α and Spliced XBP-1) (Figure 3F). In addition to RAECs, PARP16 knock-down also decreased the expression of ER stress markers (p-PERK and Spliced XBP-1) in Ang II-treated VSMCs (Supplementary Figure 1C). Immunofluorescence analysis also confirmed that co-expression levels of PARP16 and p21 were reduced in Human Umbilical Vein Endothelial Cells (HUVECs) treated with PARP16 siRNA or EGCG in response to Ang II (Supplementary Figure 3). These results indicate that PARP16 is a novel regulator of Ang II-induced vascular senescence, likely through ER stress mediated UPR.

### Binding of Smyd3 to both promotors of *Parp16* transcript variants leads to increased H3K4me3 level and elevated gene expression

**Figure 4:**
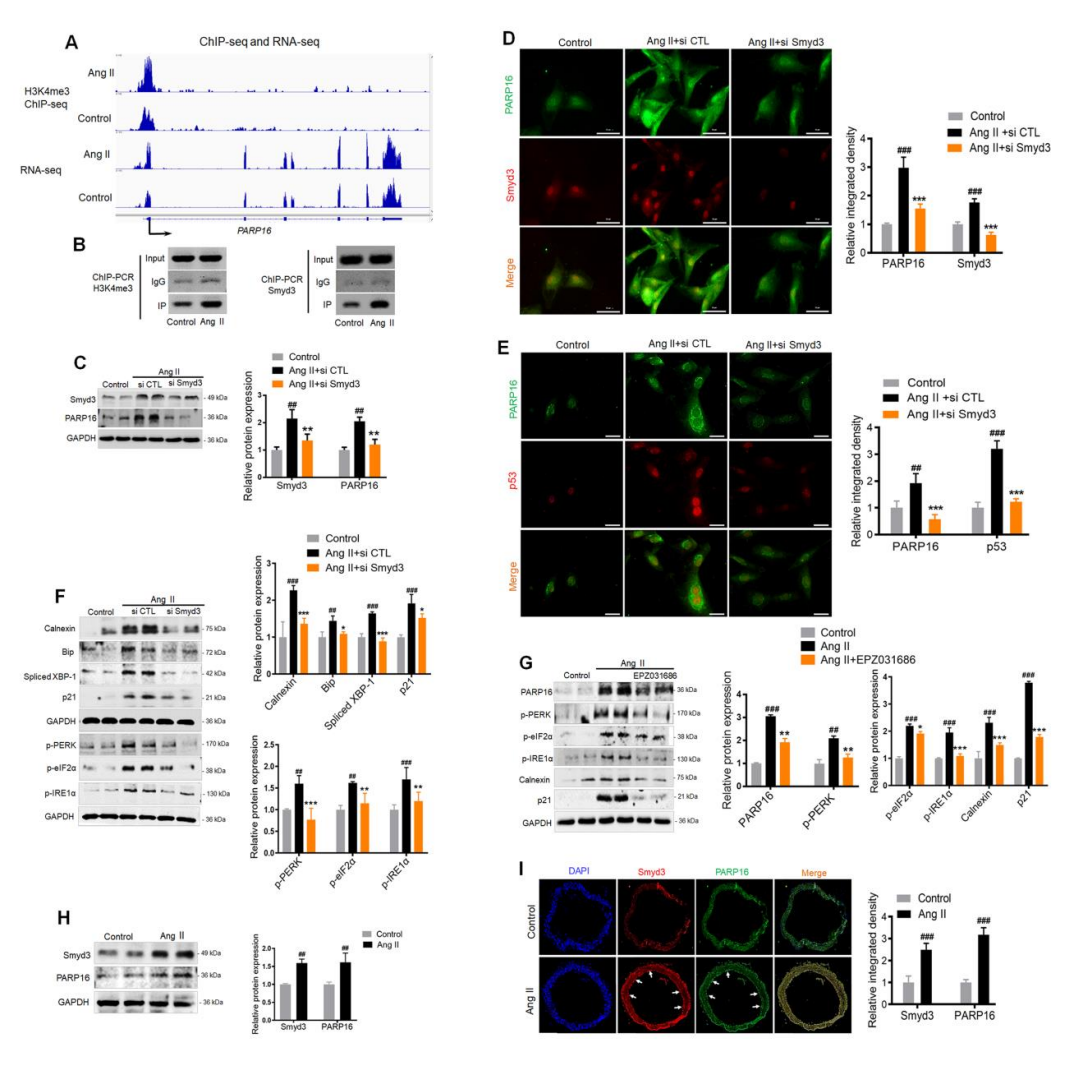
**Binding of Smyd3 to both promotors of *Parp16* transcript variants leads to increased H3K4me3 level and elevated gene expression.** (**A**) Integrative Genomics Viewer (IGV) showed signal of ChIP-seq (H3K4me3) and RNA-seq at PARP16 coding gene’s locus in control and Ang II-induced (Ang II, 48 h) RAEC cells. The Y axis was normalized to the same scale. Black arrow means primer pairs used for ChIP-PCR in panel B. (**B**) ChIP-PCR using either H3K4me3, Smyd3 or IgG antibody was carried out in control and Ang II-induced RAECs. (**C**–**F**) Knockdown of Smyd3 inhibited Ang II-induced PARP16 expression and ER stress. PARP16 was assayed by Western blot for RAECs transfected with control or Smyd3 siRNA before and after Ang II treatment. GAPDH serves as loading control (**C**); Immunofluorescence double staining of Smyd3 and PARP16 in Ang II-induced RAECs (**D**); Immunofluorescence double staining of p53 and PARP16 in Ang II-induced RAECs (**E**); ER-associated markers (Calnexin, Bip, Spliced XBP-1, p-PERK, p-eIF2α, p-IRE1α and), together with senescence-associated marker p21 were assayed by Western blot for RAEC cells transfected with control or Smyd3 siRNA before and after Ang II induction (**F**). (**G**) Smyd3 inhibitor EPZ031686 reversed Ang II-induced PARP16 and RAECs senescence. PARP16, p-PERK, p-eIF2α, p-IRE1α, Calnexin and p21 were assayed by Western blot for Ang II-induced RAEC cells with or without 20 μM EPZ031686. GAPDH serves as internal control. All data were shown as mean ± S.D of at least 4 independent experiments, ^##^*p* < 0.01, ^###^*p* < 0.001 *vs.* control; ^*^p < 0.05, ^**^p < 0.01, ^***^p < 0.001 *vs.* Ang II+si CTL or Ang II treated cells. (**H**–**I**) Smyd3 and PARP16 expression were increased in arteries from control and Ang II-infused mice model. Smyd3 and PARP16 protein expression were assayed by Western blot for aorta from control and Ang II-infused mice (**H**); Immunofluorescence double staining of PARP16 and Smyd3 of arteries in control and Ang II-infused mice model, the arrowheads indicate the positive endothelial cells staining in the whole blood vessel (**I**); Data were shown as mean ± S.D, ^##^*p* < 0.01, ^###^*p* < 0.001 *vs.* control, n=6/group.

The direct upstream regulator of PARP16 was next examined. As dramatic epigenetic changes occur during multiple cellular senescence models [23], we speculate that histone modification enzymes might regulate the PARP16 expression. Our previous study found that Smyd3, a lysine methyltransferase, induced vascular cell senescence-associated phenotypes. The DNA binding domain of Smyd3 specifically recognize 5'-CCCTCC-3' or 5'-CCCCTC-3' while the enzymatic domain dimethylates and trimethylates histone H3 at lysine 4 (H3K4) [24, 25]. Interestingly, the promotor region (2 kilobase upstream of transcription start site, or TSS) of PARP16 contains 6 potential Smyd3 binding sites (Supplementary Figure 4). An apparent increase of H3K4me3 signal was shown near the annotated TSS of *Parp16* gene in Ang II-induced cells compared to control cells by ChIP-seq, consistent with Smyd3’s methyltransferase activity on H3K4 (Figure 4A). RNA-seq data further determined that H3K4me3 abundance at the promoter region of *Parp16* upon Ang II treatment. In addition, ChIP-PCR confirmed the direct binding of Smyd3 to *Parp*’s promotor regions (Figure 4B). Further, the increase of H3K4me3 levels near the promotor of *Parp16* was validated by ChIP-PCR (Figure 4B). These data indicate that Smyd3 transcriptionally promotes *Parp16* by epigenetic modification.

To confirm whether Smyd3-PARP16 is a key axis in mediating Ang II induced ER stress, we used siRNA targeting Smyd3 to knock down of Smyd3 expression, and found blocked Smyd3 decreased PARP16 expression in Ang II-induced endothelial cells (Figure 4C). Immunofluorescence double staining showed that the co-expression of Smyd3 and PARP16 as well as PARP16 and p53 induced by Ang II disappeared upon knockdown of Smyd3 (Figure 4D, 4E). Further, silencing Smyd3 reversed Ang II-induced ER stress markers including Calnexin, Bip, p-PERK, p-eIF2α, p-IRE1α and Spliced XBP-1, together with senescence-associated marker p21 (Figure 4F). Smyd3 inhibitor EPZ031686 was also used to test whether reduced Smyd3 activity may reverse increased expression of PARP16. Excitingly, inhibition of Smyd3 could downregulate protein level of PARP16, together with reduced ER stress and senescence markers (Figure 4G). To address whether such association exists *in vivo*, mice were subjected to a 28-day infusion of vehicle or Ang II, and the aortic great vessels were assessed for Smyd3 and PARP16 expression. Consistent with *in vitro* study, the abundance of Smyd3 and PARP16 was dramatically increased in response to Ang II administration (Figure 4H). Immunofluorescence double staining showed that the expressions of Smyd3 and PARP16 in both vascular wall and inner layer where endothelial cells locate were increased in Ang II-infused mice (Figure 4I). All the above data illustrate that Smyd3-PARP16 axis is a novel signaling in mediating Ang II induced ER stress and senescence.

### Smyd3 overexpression induces PARP16 mediating ER stress

**Figure 5:**
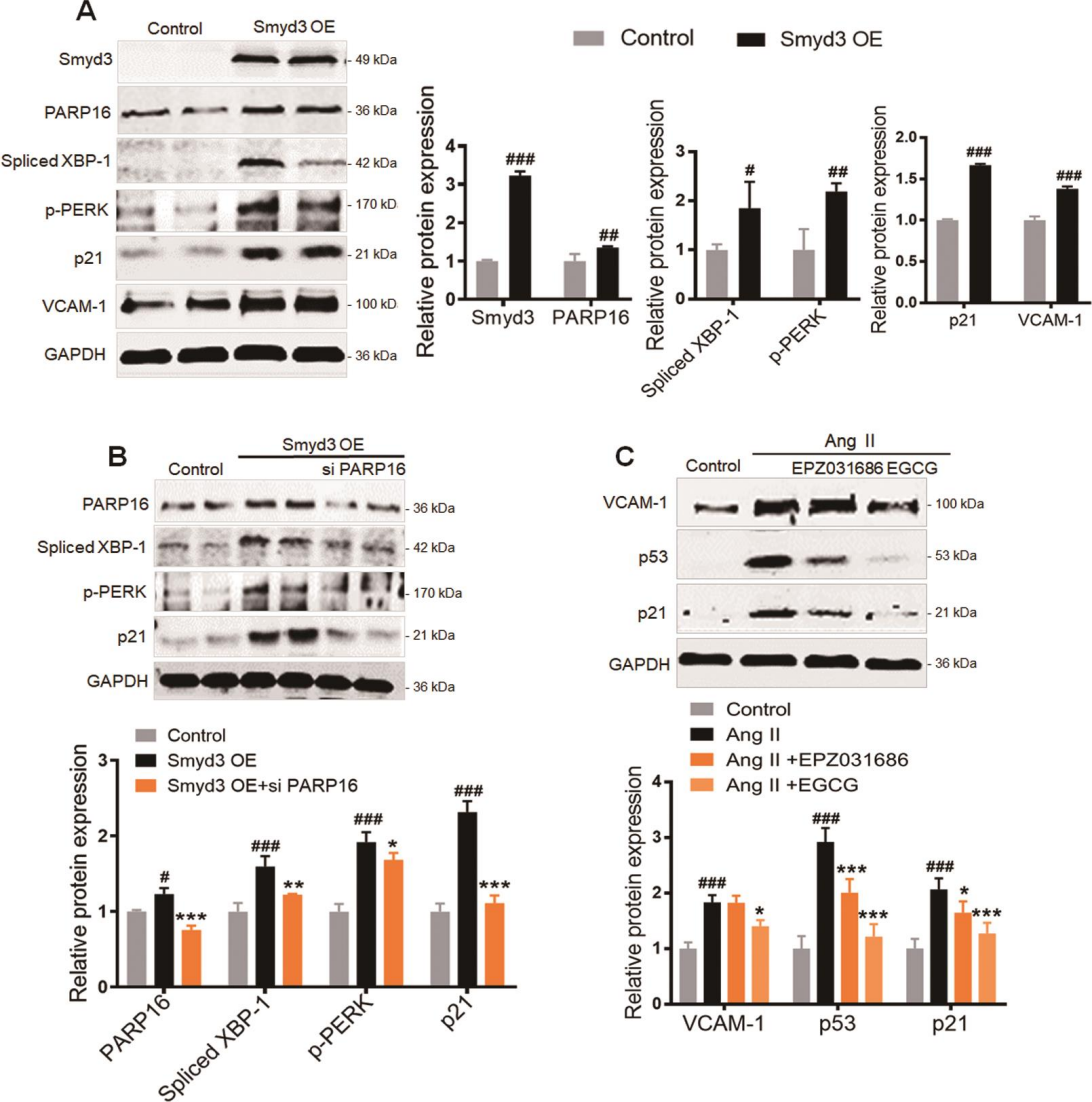
**Smyd3 overexpression induces PARP16-mediating ER stress.** (**A**) RAECs were transfected with lentivirus-mediated Smyd3 cDNA (Smyd3 OE) for 72 h, cell lysates were immunoblotted with antibody against Smyd3, PARP16, p21, VCAM-1, p-PERK, Spliced XBP-1. All data were shown as mean ± S.D of at least 4 independent experiments. ^#^*p* < 0.05, ^##^*p* < 0.01, ^###^*p* < 0.001 *vs.* control. (**B**) Knockdown of PARP16 blocked ER stress and the upregulation of p21 upon overexpression of Smyd3. Smyd3 overexpressed RAECs were transfected with control or Smyd3 siRNA, and then induced by Ang II, cell extracts were collected for determining the protein levels of PARP16, p-PERK, Spliced XBP-1, and p21 by Western blot. Data were shown as mean ± S.D of at least 4 independent experiments. ^#^*p* < 0.05, ^###^*p* < 0.001 *vs.* control; ^*^p < 0.05, ^**^p < 0.01, ^***^p < 0.001 *vs.* Smyd3 OE cells. (**C**) Inhibition of Smyd3 or PARP16 decreased RAECs senescence markers. Pretreated with PARP16 inhibitor (EGCG) or Smyd3 inhibitor (EPZ031686) for 4 h, RAECs were treated with Ang II for 48 h, respectively, cell extracts were collected for determining the protein levels of p21, p53, and VCAM-1 by Western blot. All data were shown as mean ± S.D of at least 4 independent experiments. ^###^*p* < 0.001 *vs.* control; ^*^p < 0.05, ^***^p < 0.001 *vs.* Ang II treated cells.

To further determine the higher level of Smyd3 alone promotes PARP16 expression, we overexpressed Smyd3 in RAEC cells. Intriguingly, the protein level of PARP16 was significantly increased when Smyd3 overexpressed in the endothelial cells, accompanied with elevated senescence markers such as p21 and VCAM-1 (Figure 5A). Meanwhile, increased ER stress markers including p-PERK and Spliced XBP-1 were also observed in Smyd3 overexpressing cells (Figure 5A). To examine whether Smyd3-mediated PARP16 upregulation plays a key role in senescence and ER stress, we knocked down PARP16 expression in Smyd3 overexpressed cells. Interestingly, knockdown of PARP16 blocked the upregulation of senescent marker p21 and ER stress markers upon overexpression of Smyd3 (Figure 5B). Further, both Smyd3 and PARP16 inhibitor reversed senescence-related markers (p53, p21 and VCAM-1) in various degrees (Figure 5C). These data indicate that Smyd3-driven PARP16 upregulation contributes to the senescence and ER stress.

### Knocking down or inhibiting of PARP16 prevents vascular aging in Ang II-infusion mice

**Figure 6:**
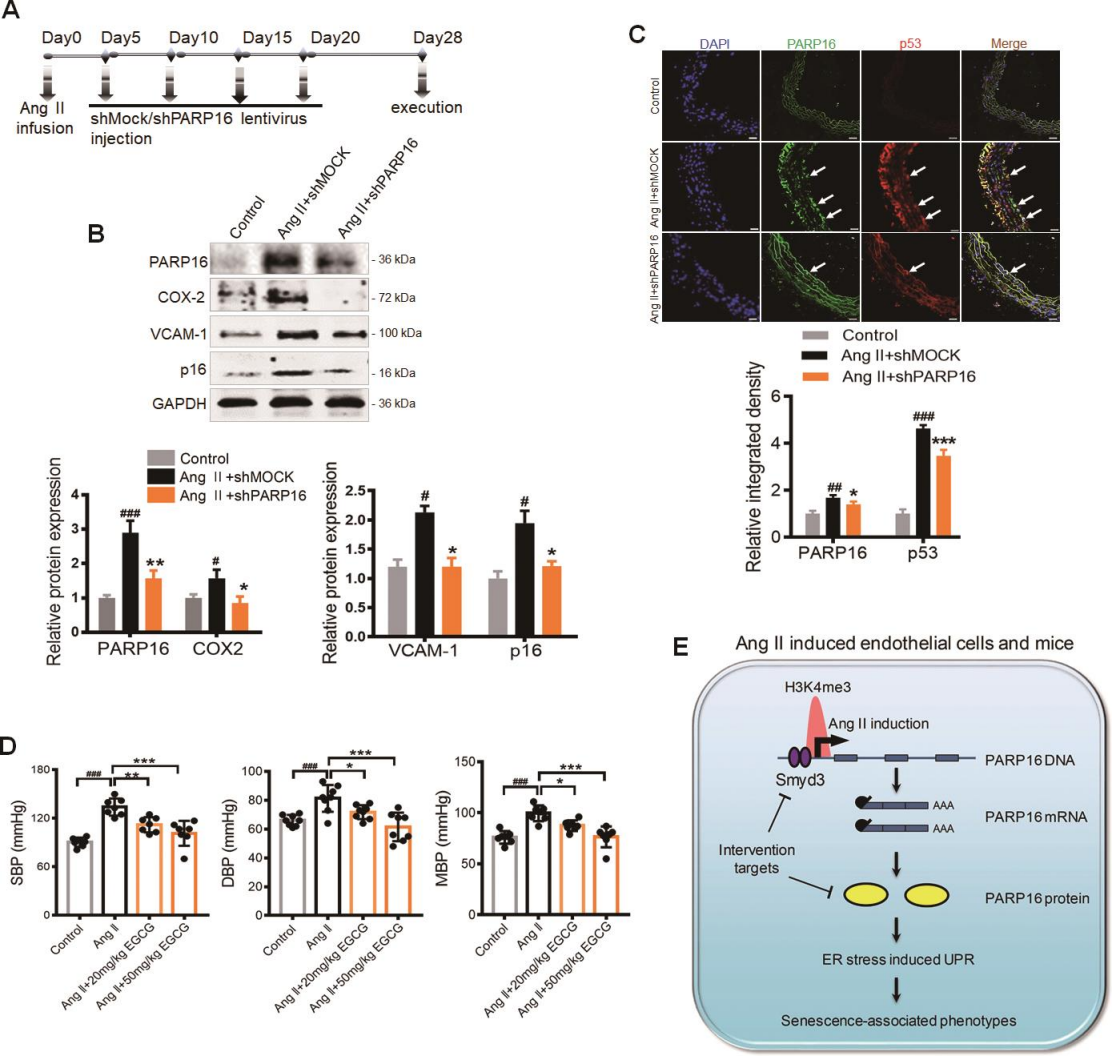
**Knocking down or inhibition of PARP16 prevents vascular aging in Ang II-infusion mice.** (**A**–**D**) PARP16 knockdown prevented vascular aging in Ang II-infusion mice. Mice received injection of either 150 μl lentivirus sh PARP16 (shPARP16) or scramble (shMOCK) every 5 days after the Ang II-infusion with mini-osmotic pumps (**A**); PARP16, p16, COX-2, and VCAM-1 proteins level were assayed by Western blot in arteries from mouse without Ang II infusion (control), Ang II-infused mouse transfected by control vector (Ang II + shMOCK) and Ang II-infused mouse transfected by shRNA of PARP16 (Ang II + shPARP16). GAPDH serves as internal control. (**B**); Immunofluorescence double staining of PARP16 and p53 in aortic great vessels of control, Ang II + shMOCK and Ang II + shPARP16 mice, the arrowheads indicate the positive endothelial cells staining in the whole blood vessel (**C**); All data were shown as mean ± S.D; ^#^ *p* < 0.05, ^##^ *p* < 0.01, ^###^*p* < 0.001 *vs.* control; ^*^p < 0.05, ^**^p < 0.01, ^***^p < 0.01 *vs.* Ang II + shMOCK; n=6/group. (**D**) EGCG attenuated blood pressure in Ang II-infusion mice. The blood pressure was assayed by the standard tail-cuff methods in mouse without Ang II infusion (control), Ang II-infused mouse, and Ang II-infused mouse with EGCG treatment. ^###^*p* < 0.001 *vs.* control; ^*^p < 0.05, ^**^p < 0.01, ^***^p < 0.001 *vs.* Ang II-infused mouse; n=7-8/group. (**E**) Working model for Smyd3-PARP16 axis accelerates unfolded protein response and vascular senescence. Younger cell has lower level of PARP16 protein and ER stress marks. In senescent cells including Ang II-induced cell, Ang II-infused mice, replicative senescence, PARP16 protein level was increased, leading to unfolded protein response and vascular senescence-associated phenotypes. PARP16 is upregulated at least partly through increased histone methyltransferase Smyd3 in senescence cells, leading to higher H3K4me3 mark at the promoter of *Parp16* gene and generating more PARP16 expression.

Given the importance of PARP16 in regulating ER stress and endothelial senescence, we hypothesized that intervention of PARP16 could be a potential strategy for preventing vascular aging. To test this hypothesis, we first knocked down PARP16 in Ang II-infusion mice model. Mice were subjected to a 28-day infusion of vehicle or Ang II, followed by treatment of lentivirus containing control (shMOCK) or PARP16 short hairpin RNA (shPARP16) (Figure 6A). The aorta and great vessels were assessed for senescence-associated phenotypes. Western blotting confirmed that PARP16 in mouse arteries could be successfully down-regulated by lentivirus *in vivo* (Figure 6B). After a 4-week treatment, PARP16 knockdown significantly alleviated the senescence-related phenotypes, including reduced expression of p16, VCAM-1, and COX2 in arteries of Ang II infusion mice (Figure 6B). Furthermore, immunofluorescence analysis of the cross-sectional area of blood vessels revealed that treatment with PARP16-targeting shRNA resulted in decreased PARP16 and p53 protein expression (Figure 6C). We also assessed PARP16 inhibitor EGCG benefit to Ang II-induced vascular aging mice model. Excitingly, the addition of EGCG reduced the elevated blood pressure caused by Ang II (Figure 6D), accompanied with decreased expression of SASP related markers including iNOS and VCAM-1 in arteries of Ang II infusion mice (Supplementary Figure 5). These data support the notion that intervention of PARP16 ameliorates Ang II-induced vascular senescence in mice.

### PARP16 upregulation exists also in cellular replicated senescence models

Replicative senescence induces vascular cell growth arrest and loss of vascular homeostasis, contributing to the initiation and progression of cardiovascular diseases, we further examined the dynamic expression changes of PARP16 in replicative senescence of RAEC. Western blot showed an increase of PARP16 in RAECs undergoing replicative senescence (Supplementary Figure 6A). ER stress markers were also detected in replicative senescent RAECs (Supplementary Figure 6B). Together, higher expression of PARP16 exists in replicative senescence, highlighting the potential biomedical importance of understanding and intervention of such regulation mechanism.

## DISCUSSION

PARP16, a tail-anchored protein located at the endoplasmic reticulum membrane, is required for activation of the functionally related ER stress sensors PERK and IRE1 during the UPR. Here we have identified that PARP16 promotes vascular cell senescence and ER stress, and blocking PARP16 significantly alleviated the senescence-associated phenotypes in Ang II-treated mice and vascular cell. Further, PARP16 was epigenetically upregulated by Smyd3, a histone H3 lysine 4 methyltransferase that bound to promotor of PARP16 and increased H3K4me3 level to activate its host gene’s transcription. Finally, Smyd3-PARP16 signal axis mediated senescence and ER stress exist in cell models (Ang II-induced premature aging and replicative senescence), animal models (Ang II-infused mouse).

Aging is one of the causes of various cardiovascular diseases and perturbs a number of metabolic and hemodynamic mechanisms in the cardiovascular system in general and the vascular endothelium in particular [26, 27]. Proteostasis impairment and ER stress are proposed as one of the hallmarks of senescence and contribute to the development of aging-related diseases. Recent evidence places ER stress as a driver of brain aging, and the emerging impact of neuronal UPR in controlling global proteostasis at the whole organismal level. In addition, ER stress has also been detected in endothelial cells subjected to atherosclerosis-prone shear stress [28]. Although it is clear that ER stress and activation of the UPR are components of the senescent phenotype, whether ER stress and the UPR are cause or consequence of cell senescence is largely unknown, this could be due to the diversity in the UPR signature and the versatility in the activation of the three UPR branches. It is reported recently that an overactivity of the ER could be a source for senescence-associated oxidative stress [29] and the UPR could also participate in the induction of the senescent cell-cycle arrest pathway. Ang II, a major hormone, is significantly increased in pathological state, and could lead mice to develop age-related cardiovascular diseases such as high blood pressure, cardiac hypertrophy and fibrosis. Consistent with the report, we demonstrated that Ang II-induced the appearance of aging phenotype, along with ER stress including PERK, IRE1α and ATF6 three branches pathways in both cell and mice models. In our study, although apoptotic arm-CHOP mRNA levels were upregulated in Ang II treated RAECs, but some other apoptotic arm genes including *PUMA*, *p53*, *NOXA*, *Bax* and cell viability were not changed obviously after Ang II treatment (data not shown). Further, PARP16, a tail-anchored ER transmembrane protein required for activation of the functionally related ER stress. PARP16 has been reported as an effective activator of ER stress sensor PERK and IRE1α through increasing their kinase activities and endonuclease activity of IRE1α. In this study, we have demonstrated PARP16 is involved in Ang II-induced vascular senescence. On contrary, knocking down of PARP16 led to reversed senescence-related phenotypes including cell-cycle arrest and β-Gal staining, at least partly through mediating PERK and IRE1α branches of ER stress. EGCG has been reported is a potential inhibitor of PARP16, which suppressed the ER stress-induced p-PERK and the transcription of UPR-related genes. We also showed that EGCG reversed senescence-related phenotypes and raised blood pressure caused by Ang II. These results indicated that agents targeting PARP16, such as EGCG or gene intervention probably provide new ideas for clinic prevention of age-related cardiovascular diseases.

In our previous study, we found Smyd3, an epigenetic writer, was increased in vascular ageing. The expression of Smyd3 was up-regulated in Ang II-induced vascular senescence and knocking down of Smyd3 reversed senescence-related phenotypes and elevated blood pressure. In this study, we further found knocking down of Smyd3 significantly depleted PARP16 expression along with the reduced expression of ER stress and senescence-related markers, which stated that Smyd3 knockdown may inhibit ER stress through suppressing PARP16 expression. Besides, the consistent results could be drawn from the application of Smyd3 specific inhibitor. Previous studies indicated that Smyd3 was elevated in cancer-associated phenotypes through methylation of histones (H3K4, H4K5 and H2A.Z) and non-histone proteins [30]. In addition, Smyd3 specifically recognize 5'-CCCTCC-3' or 5'-CCCCTC-3' while the enzymatic domain dimethylates and trimethylates histone H3 at lysine 4 (H3K4), to activate the transcription of multiple target genes [31, 32]. These observations led us to investigate whether the H3K4 methylation dependent function of Smyd3 could increase the transcription level of PARP16. Interestingly, we found H3K4me3 has higher abundance at promotor regions of PARP16 in Ang II-induced senescent endothelial cells by ChIP-seq, supporting this hypothesis, genes with higher level of H3K4me3 increased mRNA expression. RNA-seq data further determined that H3K4me3 abundance at the promoter region often positively correlates with PARP16 expression levels. Meanwhile, the promotor region of PARP16 contains 6 potential Smyd3 binding sites, ChIP analysis confirmed the direct binding of Smyd3 to *Parp16*’s promotor regions, and transcriptionally activated PARP16 to promote the appearance of aging phenotype, providing a previous unestablished association between Smyd3 and PARP16.

The activation of the UPR seems to occur in all types of senescence, whether the inducer is successive replications [33], oncogene activation [34, 35], DNA-damaging agents such as X-rays [36] or Adriamycin [35], or oxidative stress [37]. Indeed, a recent article shows that stress-induced senescence can be promoted through the activation of an ER stress-dependent p21 signaling [38]. The three branches are not always activated together in a given senescence context, and none of them seem specific for a type of senescence [8]. Replicative senescence caused vascular cell growth arrest and loss of vascular homeostasis, also contributing to the initiation and progression of cardiovascular diseases. We found higher expression of Smyd3-PARP16 signal axis also exists in replicative senescence, suggesting that Smyd3-driven PARP16 upregulation may play a key role in vascular senescence and ER stress.

In this study, we firstly found PARP16, a tail-anchored ER transmembrane protein, played a novel and key role in promoting vascular senescence, likely through regulating the unfolded protein response of ER. Further, PARP16 was upregulated by Smyd3, which bound to promotor of *Parp16* and increased H3K4me3 level and ultimately activated its transcription. To the best of our knowledge, we discovered Smyd3-PARP16 signal axis as a novel and important factor in regulating Ang II-induced endothelial senescence and function disorder. Such mechanism may also exist in replicative senescence model and also in Ang II infusion mice model (see working model in Figure 6E). Intervention of either Smyd3 or PARP16 could ameliorate vascular aging associated phenotypes, highlighting the implications in preventing vascular aging and related diseases.

## MATERIALS AND METHODS

### Materials

Reagent sources were as follows: angiotensin II (Ang II), epigallocatechin gallate (EGCG, a PARP16 inhibitor), 4μ8C (a specific IRE1 inhibitor) and ISRIB (a specific PERK inhibitor) were purchased from Meilunbio (Meilun Biotechnology, Dalian, China). EPZ031686 was purchased from MCE (MedChemExpress, USA). Antibodies were obtained from the following commercial sources: PARP16, Smyd3, H3K4me3, Bip and XBP-1 were purchased from Abcam; VCAM-1, Calnexin, and GAPDH was purchased from Proteintech (Proteintech, USA); IL-6 and iNOS were purchased from Epitomics (Burlingame, CA); p-PERK, p-eIF2α, p53, p21 and p16 were purchased from Cell Signaling Biotechnology (Danvers, MA, USA); p-IRE1α were purchased from Thermo Fisher (MD, USA); ATF6 was purchased from Bioss (Bioss Antibodies, Beijing, China).

### Animal experiments

C57BL/6 mice were randomly assigned to one of three groups: The sham control group, Ang II-infusion group or shRNA PARP16 group (KD PARP16). Mice were anesthetized with isoflurane, followed by subcutaneous implantation of an Alzet osmotic minipump (Model 2004, ALZA Scientific Products, Mountain View, CA, USA) containing only saline, or Ang II (Meilun Biotechnology, Dalian, China) dissolved in saline. Mice were then continuously infused with saline or Ang II (1.5 mg/kg/day) for 4 weeks. Infusion 5 days later, KD PARP16 group and Ang II-infusion group were injected with lentiviral vector expressing shRNA PARP16 and shRNA scramble (sh MOCK) *via* their tail veins after, respectively. In total, 4 injections were administered, once every 5 days, each injection contained 0.15 mL of the concentrated viral suspension with a titer of 1×10^8^ IU/mL.

For EGCG groups, after mice were subcutaneously implanted with osmotic minipump, EGCG at doses of 20 and 50 mg/kg/day was administered to mice by intraperitoneal injection for 4 weeks.

C57BL/6 mice were sacrificed by cervical dislocation and the thoracic aorta of each mouse was cut into two sections. One section was stored at -80°C for protein extraction. The other section was fixed with 4% paraformaldehyde overnight, and was then embedded in paraffin for immunofluorescence staining. Paraffin-embedded sections (8 μm thick) were cut every 200 μm length of thoracic aortas from the proximal thoracic aortas specimens.

### Culture of rat primary aorta endothelial cells (RAECs)

RAECs were obtained as our described [39]. Cells that experienced 3-5 passages were used for Ang II-induced senescence experiments performed in this manuscript.

For the replicative senescence, the cells then underwent serial passaging to reach senescence. The senescent status was verified by staining for SA-β-gal. 90% percent of the cells at 10 passages stained positive for SA-β-gal.

### shRNA and overexpression lentivirus generation and infection

The sequences of shRNA targeting mouse *Parp16* gene were as follows: mouse_*Parp16*_shRNA1_F: 5'-CCGGCAAGTGCCAAATCAAGAAGAACTCGAGTTCTTCTTGATTTGGCACTTGTTTTTG-3'; mouse_*Parp16*_shRNA1_R: 5'-AATTCAAAAACAAGTGCCAAATCAAGAAGAACTCGAGTTCTTCTTGATTTGGCACTTG-3'; mouse_*Parp16*_shRNA2_F: 5'-CCGGCCTGAACAAGACTTCTCTGTTCTCGAGAACAGAGAAGTCTTGTTCAGGTTTTTG-3'; mouse_*Parp16*_shRNA2_R: 5'-AATTCAAAAACCTGAACAAGACTTCTCTGTTCTCGAGAACAGAGAAGTCTTGTTCAGG-3'. The oligos contain the shRNA sequence flanked by sequences that are compatible with the sticky ends of EcoR I (NEB) and Age I (NEB). Forward and reverse oligos are annealed. Then pLKO.1 TRC Cloning Vector (Addgene) was digested with restriction endonucleases EcoR I and Age I. After that, the PARP16 insert and linearized plasmid were joined by T4-DNA ligase (NEB) and ligated into the pLKO.1 TRC Cloning Vector, producing a final plasmid that expresses the PARP16 shRNA.

For PARP16 overexpression (PARP16 OE) and Smyd3 overexpression (Smyd3 OE) plasmid construction, the rat genomic DNA was used to amplify the PARP16 and Smyd3 full-length cDNA, then cloned into the vector pCDH-CMV-MCS-EF1-copGFP. The product was confirmed by sanger sequencing.

To obtain the lentivirus, the recombinant plasmid and packaging vector psPAX2 and PMD2.G were co-transfected into 293T cells using transfection reagent lipofectamine 2000 (Invitrogen, USA). After incubation for 48 h, the lentivirus in the culture medium was collected by filtration with 0.45 μm filters and then concentrated *via* ultracentrifugation for 2 h at 250,000 rpm. The viral particles were resuspended in PBS and stored at -80°C. Lentivirus encoding PARP16, Smyd3 or control vectors in the presence of 8 μg/ml polybrene (Sigma) was added to RAECs culture media for 24 h. This medium was then removed and the cells were allowed to incubate for additional 48 h under normal culture conditions.

### Small interfering RNA (siRNA) transfection

Rat PARP16, Smyd3, and control siRNA were produced by GenePharma (Shanghai, China). The sequence of the siRNA was as follows: PARP16: 5’-CCUACCUCACAAGUGACUUTT-3’; and 5’-AAGUCACUUGUGAGGUAGGTT-3’. Smyd3: 5'-CUGAUGCGGUGUUCUCAAUTT-3', and 5'-AUUGAGAACACCGCAUCAGTT-3'. Negative control: 5’-UUCUCCGAACGUGUCACGUTT-3’. To introduce siRNA into RAECs, the cells were plated on 6-well plates at 30% to 50% confluence before transfection. Individual siRNAs (15 nM), Lipofectamine RNAiMAX, and Opti-MEM were mixed and incubated at room temperature for 5 min. siRNA-lipofectamine RNAiMAX complexes were added to cells and incubated for 24 h, then the medium was replaced with fresh serum DMEM medium. Experiments were performed 72 h after transfection.

### RAECs proliferation analysis

To assess cell proliferation, immunofluorescence assay was also used for the detection of EdU incorporated into cellular DNA (EdU Labeling and Detection Kit, KeyGEN BioTECH, Jiangsu, China). The immunofluorescence detection was performed according to the manufacturer's instructions. Total cellular nuclei were stained with DAPI. Immunofluorescence EdU positive cells were observed under the Zeiss inverted fluorescent microscope. And the EdU incorporation rate was expressed as the ratio of EdU positive cells to total DAPI positive cells (blue cells).

### SA-β-galactosidase staining

The expression of senescence-associated β-galactosidase (SA-β-Gal) in cells was determined by SA-β-Gal staining as described [40]. SA-β-Gal positive cells was expressed as the ratio of SA-β-Gal positive cells to total cells.

### Quantitative real-time reverse transcription polymerase chain reaction (qRT-PCR) analysis

Total RNA was extracted from RAECs with TRIzol Reagent (TaKaRa Biotechnology, Dalian, China) following the manufacturer’s instructions. Total RNA (600 ng) of each sample was reversely transcribed into cDNA using two-step RT Kit (Takara Biotechnology, Dalian, China) according to the manufacturer’s directions and Real-time qPCR was performed with Bio-Rad CFX Connect^TM^ Real-Time PCR System (Bio-Rad, Hercules, CA, USA). Primer sequences of genes are listed in the Supplementary Table 1. Level of expression was quantified by 2^−ΔΔCt^ equation, with GAPDH as control for normalization.

### RNA-seq and Chromatin Immunoprecipitation coupled with deep sequencing (ChIP-seq) library construction

PolyA+ RNA was enriched by oligo(dT)25 Dynabeads (Invitrogen) from total RNA extracted as above. The dUTP-based strand-specific RNA-seq libraries for control and Ang II induced RAEC cells were constructed according to the protocol of Parkhomchuk et al. [41]. ChIP-seq libraries were constructed according to the protocol established previously [42, 43], and H3K4me3 antibody (ab8580, Abcam) was used during the immunoprecipitation step. After quality inspection, both RNA-seq and ChIP-seq libraries were sequenced by Illumina HiSeq platform, and paired-end reads of 150 nt length were obtained.

### Chromatin immunoprecipitation PCR (ChIP-PCR)

Both H3K4me3 (ab8580, abcam) and Smyd3 antibody (GTX121945, GeneTex) were used during the immunoprecipitation step before PCR. Taq DNA Polymerase (Yeasen) was used for the PCR step, and the multiple sets of primers spanning the transcription factor binding site on *Parp16* gene promoter subjected to PCR analysis. Primer sequences are as follows: *Parp16*: 5’-CAGGACTGACTGCAGAGTGC-3’, and 5’-ACTCTGTAGCCCATGCTGAC-3’. Thermal cycling was carried out as follows: 94°C for 30 s; 30~40 cycles of 94°C for 30 s, 52~58°C for 30 s and 72°C for 30 s; 72°C for 10 min; hold at 4°C. The PCR product was visualized in a 2% agarose gel stained by Gel-Red.

### Western blotting

Samples were prepared with RIPA buffer (Pierce, Rockford, IL, USA) containing protease and phosphatase inhibitor cocktail (Sigma, St Louis, USA). Whole lysates samples were separated by SDS-PAGE and blotted to nitrocellulose membrane. Protein bands were detected with fluorophore-conjugated secondary antibodies, and detection and analysis were performed with the Odyssey imaging system (LI-COR).

### Immunofluorescence staining

Cells were seeded on glass coverslips placed in 24-well plates. Cells were fixed with 4% paraformaldehyde for 15 min, followed by permeabilization with 0.25% Triton X-100 in PBS 10 min. Next, the slides were blocked in PBS with 10% goat serum for 30 min, and incubated overnight with primary antibodies at 4°C. Appropriate secondary antibodies were added and incubated with cells for 1.5 h at room temperature. The nuclei were stained with DAPI. The images were captured by using a fluorescence microscope (Axio Scope.A1, Carl Zeiss Imaging Systems).

### Statistical analysis

Statistical analysis was performed using the software GraphPad Prism version 7.0. All values are expressed as the mean ± S.D. One-way ANOVA were initially performed to determine whether an overall statistically significant change existed before using the two-tailed paired or unpaired Student’s t-test. For each test, *p* values less than 0.05 were considered significant.

### Ethics statement

Male C57BL/6 mice (22-25 g) were purchased from SHANGHAI SLAC LABORATORY ANIMAL CO. LTD (Shanghai, China). All animals were housed under conventional conditions in the animal care facilities and received humane care in compliance with the Principles of Laboratory Animal Care formulated by the National Society for Medical Research and the Guide for the Care and Use of Laboratory Animals. The experimental protocol conformed to the Animal Welfare Act Guide for Use and Care of Laboratory Animals, and was approved by Institutional Animal Care and Use Committee (IACUC), School of Pharmacy, Fudan University, China.

## Supplementary Material

Supplementary Figures

Supplementary Table 1
