# Supplementary material for: Smyd3-PARP16 axis accelerates unfolded protein response and vascular aging
Source: Aging (Albany NY). 2020 Nov 3;12(21):21423–45. doi: 10.18632/aging.103895 (PMC7695420; doi:10.18632/aging.103895)
Supplement: Supplementary Figures [file aging-12-103895-s001..pdf]

SUPPLEMENTARY FIGURES

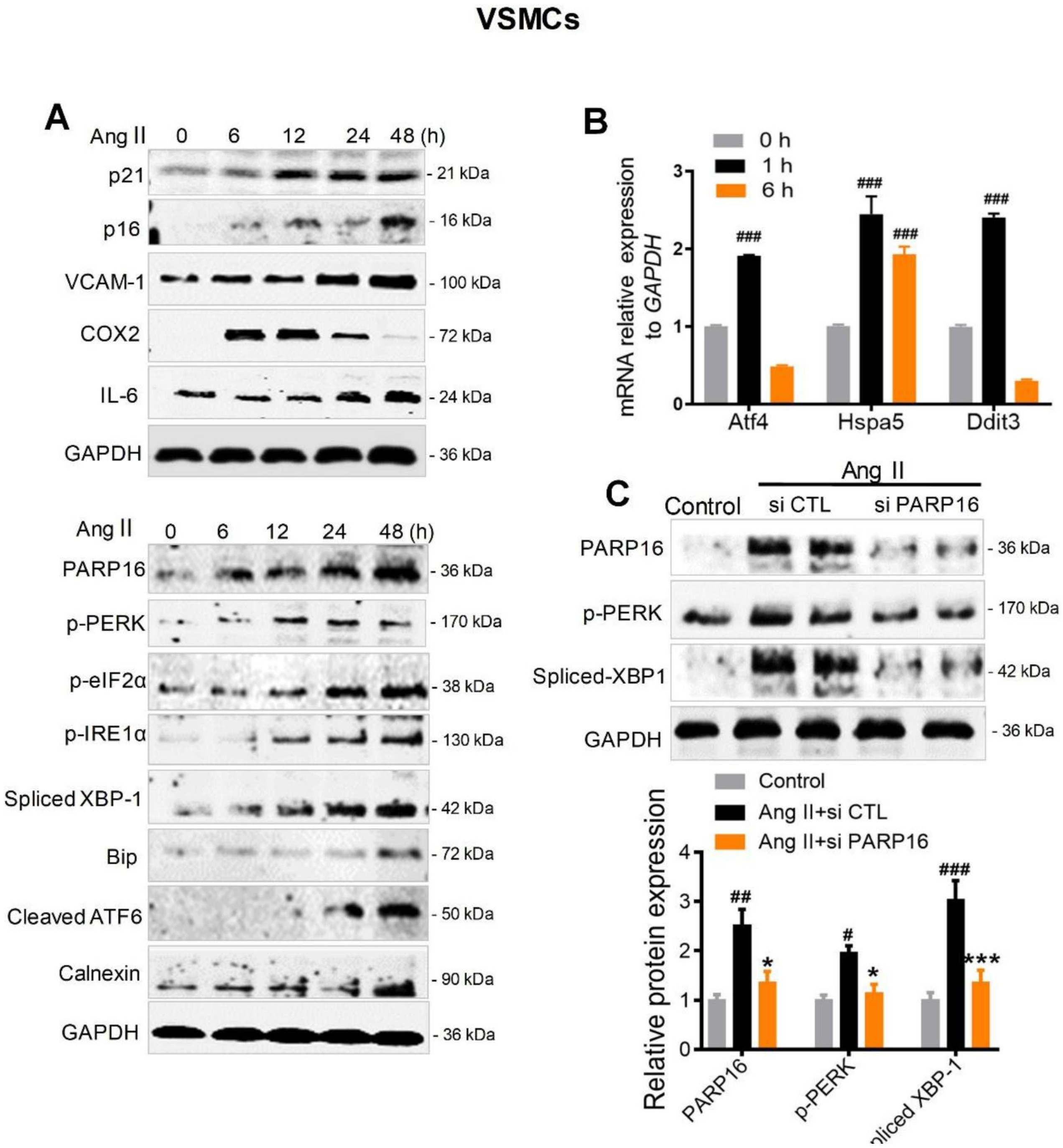

Supplementary Figure 1. PARP16 is involved in Ang II-induced VSMCs and HUVECs ER stress and senescence. (**A**) Senescence-associated markers, ER markers and PARP16 expression are upregulated after Ang II stimulation in VSMCs. At 0, 6, 12, 24 and 48 h after Ang II (2 μM) administration, cells extracts were collected for determining the protein levels. (**B**) the UPR target genes (*Atf4, Hspa5, Ddit3*) is increased by Ang II treatment in VSMCs. (**C**) p-PERK, spliced XBP-1 were assayed by Western blot for VSMCs transfected with scramble (si CTL) or PARP16 siRNA before and after Ang II induction. Protein quantitative analysis was shown at the bottom of each Western blot. GAPDH serves as internal control, $^{\#}p < 0.05$, $^{\#\#}p < 0.01$, $^{\#\#\#}p < 0.001$ *vs.* control; $^{*}p < 0.05$, $^{***}p < 0.001$ *vs.* Ang II+siCTL treated cells; all data were shown as mean ± S.D of at least four different replicates.

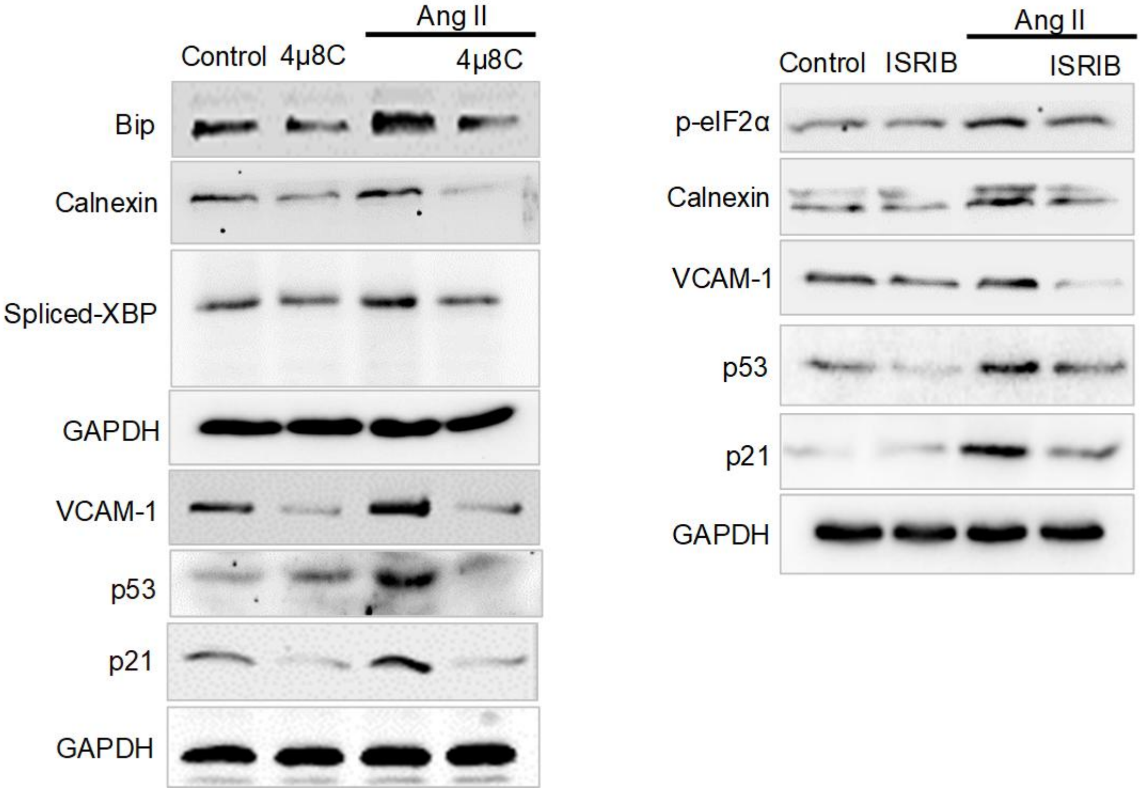

Supplementary Figure 2. Inhibitor of IRE1 or PERK signaling decreases the expressions of Ang II-induced RAECs senescence markers. RAECs were treated with Ang II (2 μM) for 48 h in the presence of 4μ8C (50 μM), ISRIB (1 μM) or DMSO vehicle, Bip, Calnexin, Spliced-XBP-1, p-eIF2α, p53, p21 and VCAM-1 levels were detected by Western Blot, respectively. Data shown are represent data from three independent experiments.

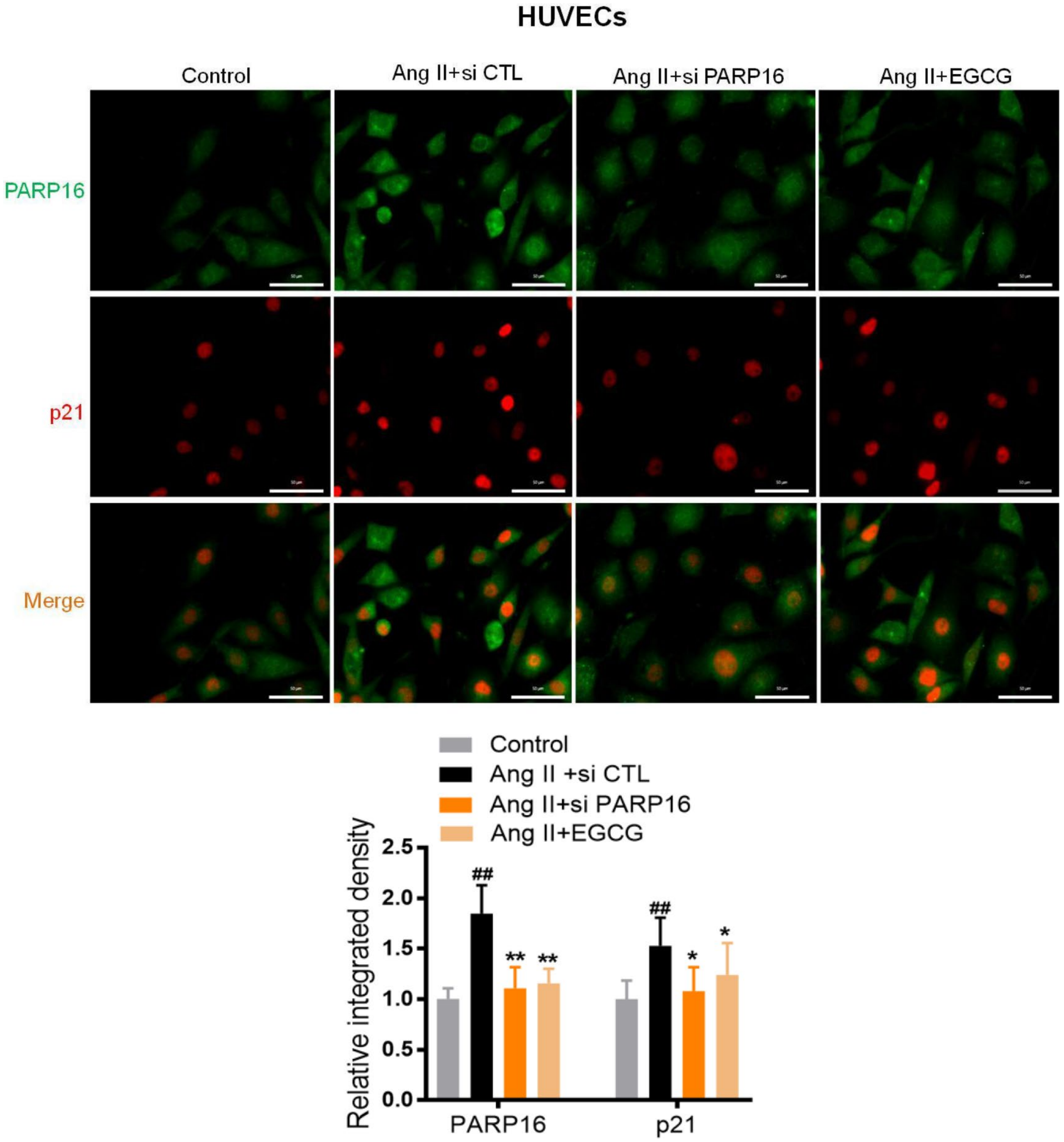

Supplementary Figure 3. Immunofluorescence double staining of PARP16 and p21 for HUVEC cells transfected with PARP16 siRNA or treated with PARP16 inhibitor (EGCG) before and after Ang II induction. Data were shown as mean ± S.D of at least four different replicates; $^{\#\#}p < 0.01$ *vs.* control; $^{*}p < 0.05$, $^{**}p < 0.01$ *vs.* Ang II+siCTL treated cells.

PARP16 gene TSS upstream 2000bp

\>rn6_refGene_NM_001014093 range=chr8:70710780-70712779 5'pad=0 3'pad=0 strand=+
repeatMasking=none

TATATAGTGAGAACTTTTCTAAGCAACAATAAACCAAAACCAACCAAAAACCAACCAAATAACAAC
CAACTAACCAAACAAACAAGAAACCCCCACTGTTATGCCATTACTGTGGGTATACTCCCTGCAG
TCATGGTGAGCATACCCCTGCAGTCACTGTGGATGTACCCCTTTCTGGGTGATAAGAGTGGCAA
CTTCAGAGGCTCTGAAGGGATGAGATGGACTCGATTCCCTCTACTGACATTACTAAATAAACAAA
CAAGCAGACAGTAATAACACACCCCAGAAATGGGAGGACAAGCAATAGTCTATCCTCTAAAATG
CCCTTTCTCTAGAACAACAGAAAT**GAGGG**CACAGAAGCAGAATAGGACTCTCTGTGAAGAGCA
AACAGAAGGGGTAGCAGGACTGACTGCAGAGTGCGCAGAGGTCACTCTTATGAATAAGGAGC
CAAAAGAGTATGATTAAA**GGAGGG**CAGGACTGCCAAAGGAGAATGTCACATCAAATACAGCATA
TCACCGAAGAAAGAAACTGTGTTGTTTTGTCTGAGGTCAGCATGGGCTACAGAGTAAGACCAC
AGAGAAAATAAGGGAAGATTCCATACATTTTGTTGTCGTTGTTGCCAGTTGTTGTGGCACTGTG
GCATGGAGAATGCCTTTACTTCCAGCACAAAAACAAACAAACAAACAAACAAACAAACGGCGAC
AAAAATAAGTCTTCTAGGTTTCCAGGAAGCCCAATGAACTTCAAGTAGCATCAATGCAAAAGTTT
ATAAAGAGAAACAACACGGCAAAGATATGGAGAGTCAAAGACCA**GGAGGG**AATCTTTTAAATTT
AAATTTTATTATTATCTTTTTTATTTGTTTGTTTTTCAACATAGGGTAGCCCTGGCTGTCCTAGAATT
TGCTCTGTAGATCAGGCTGGCCTCAAACCCACAGAGATCAACCTGTCTCTGCCTCTTGAGTACT
GGATGAATAAATTTTAAAGATTTATTTCTTAATTTTTTTTTTTTTTGGTGAAGTTTGTTTTGTTTTGGG
ATTTTTGTTTTCGTTTTTTTGAGACAGGGTTTCTCTGTGTAGCCTTGGCTGTCCTGGAACTTGAT
CTGTAGACCAGTCGGGACTTGAACTCAGAGATCTGCCTGCCTCTGCTGGGACTAAAAGTGTGT
CATATGTGAGGTTTTTAAAGGGGGGCCTTTTGGTCTAACAATTTACCACAGGCTTAGAGTTCTGT
GATCAGTAGGCCAAATACCTAGTGAGGTC
TTGAGGAGGAAGAAGTAAAGGGAATCTGTTCTTACATTTATTTATTTTGTGTACATTCACATATGG
GCACACTTGAGCCTTGGGCATGTGTGGAAGTTATAGGACAAACTGCTGGAATCCGTTAGCTCCT
AACATGTGGGATCCCGGGATCGAGCTCAGGTCGTGAGGACTGACAGCAAGTGCCTTTACCCAT
CCGGTTGTCCAGTCGCTGAGTCATCTTGGGGTCAGGTCTTTTCCCTTCCGCAGGGTTAGGTCA
TTGTTCTCTCAGTACATGTTTTCCCGACTTCCTGTTAGAGGCCTTCGAAGGAGGAGGATCCTGG
GAAATCTGAGGTCAGACATTTGAGCAATTCAGAGAATTCCGGAGGCTGTAAGGTAGAGAGAAA
GGACTTTGGGTCAGCTACAATGTCTTGAGGAAGTATTGTGTCACTCCGACCCTCAGTTTTCCCT
GGGTGTCAAGTGGCTCCTTTTGTGCAACGTGTCAGCTCCAGAGGAAACCGACTTATTCAACAG
GTGGTAATTCCCAAGGTGAGAAAAGCAGACTCAACCGGAACAGCCAGGACAAAGACAATTCCC
CGGGGCGGATCCCGGATCGGTTT**CCTCC**TAGGGCTGTGGCGAGGATGATTAGCAGCGGCACG
GGCACCTTTGGCCCGCACCCTGGGCAGCTGGAATCCTCGGGGACGTGTGGGCCGGCGCGAG
CGGAGCTAGGGCGCGGGAAGGGGCGGGCCTGGGCCCGGTGGGCGGAGCGG

Smyd3：Binds DNA containing 5-CCCTCC-3 or 5-GAGGGG-3 sequences
5-GGAGGG-3or 5-CCCCTC-3

Supplementary Figure 4. The promotor region (2 kilobase upstream of transcription start site, or TSS) of PARP16 contains 6 potential Smyd3 binding sites

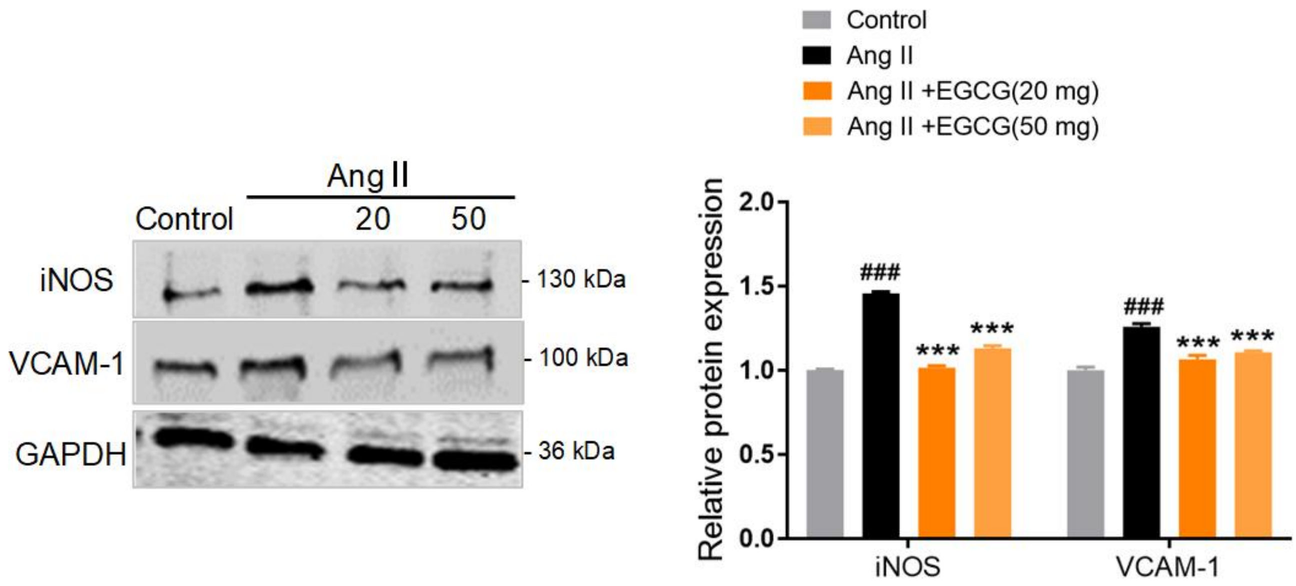

Supplementary Figure 5. ECGC decreases vascular inflammation markers in Ang II-infusion mice. Western blot of VCAM-1, iNOS, and GAPDH in aortic great vessels from mouse without Ang II infusion (control), Ang II-infused mouse and Ang II-infused mouse treated by 20, 50 mg/kg/day EGCG. $^{###}p < 0.001$ *vs.* control; $^{***}p < 0.001$ *vs.* Ang II group; all data were shown as mean ± S.D, n=6/group.

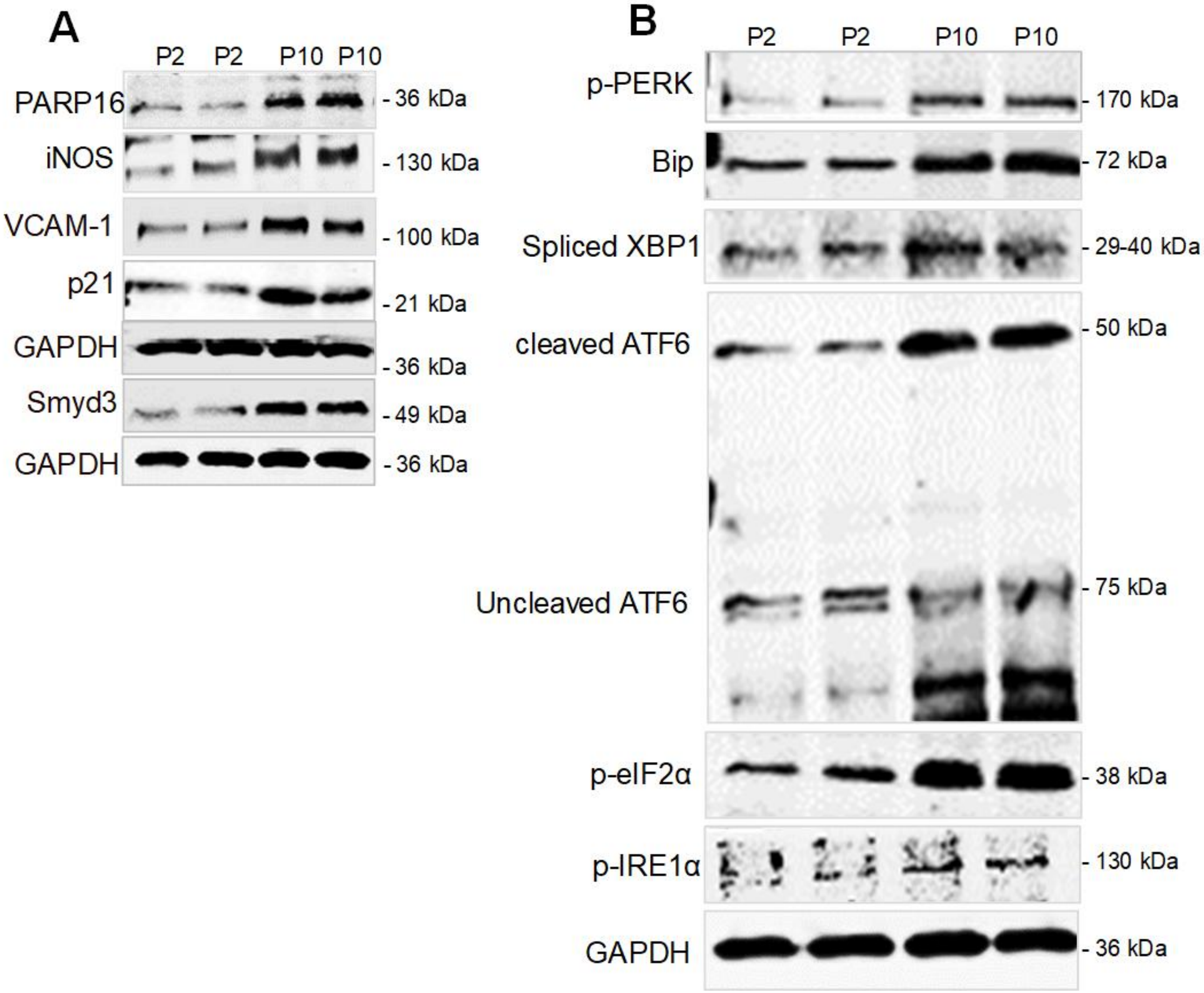

Supplementary Figure 6. PARP16 upregulation exists also in cellular replicated senescence models. (**A**) PARP16 upregulation exists in cellular replicated senescence models. Western blot of PARP16, senescence marker (p21), and SASP markers (VCAM-1, iNOS) in passage two (p2), and passage ten (p10) of replicative RAEC cells; (**B**) ER stress markers (p-eIF2α, p-IREα, p-PERK, cleaved ATF6, Bip and Spliced XBP-1) expression were determined by Western blot in passage two (p2) and passage ten (p10) of replicative RAEC cells.
