# Supplementary material for: Smyd3-PARP16 axis accelerates unfolded protein response and vascular aging
Source: Aging (Albany NY). 2020 Nov 3;12(21):21423–45. doi: 10.18632/aging.103895 (PMC7695420; doi:10.18632/aging.103895)
Supplement: Supplementary Table 1 [file aging-12-103895-s002..pdf]

SUPPLEMENTARY TABLE

Supplementary Table 1. Primers used for qRT-PCR validation.

Gene name	Primer name	Primer sequence (5′ to 3′)
Parp16	Rat _Parp16_ F	CTCCTTGGCCAGACCCTTAG
	Rat _Parp16_ R	GATAGAGGGACATCGCGACC
Cdkn1a	Rat _Cdkn1a_ F	GTGGACAGTGAGCAGTTGAG
	Rat _Cdkn1a_ R	TCAGGTAGATCTTGGGCAGC
Tp53	Rat _Tp53_ F	GACGGGACAGCTTTGAGGTT
	Rat _Tp53_ R	CTCCGGGCAATGCTCTTCTT
Il6	Rat _Il6_ F	CTCTCCGCAAGAGACTTCCA
	Rat _Il6_ R	CTCCTCTCCGGACTTGTGAA
Vcam1	Rat _Vcam1_ F	CTGCACGGTCCCTAATGTGT
	Rat _Vcam1_ R	CAAGAGCTTTCCCGGTGTCT
Nos2	Rat _Nos2_ F	CAGCCTGTGAGACGTTCGAT
	Rat _Nos2_ R	CCCATGTTGCGTTGGAAGTG
Hspa5	Rat _Bip_ F	AGTTGTGACTGTACCAGCTTACT
	Rat _Bip_ R	ACATCGAAGGTTCCACCACC
Ddit3	Rat _CHOP_ F	GCTTGCTGAAGAGAACGAGC
	Rat _CHOP_ R	GACTGACCATGCGGTCGAT
Atf4	Rat _ Atf4_ F	CTGAGTCCTACCTGGGCTCT
	Rat _ Atf4_ R	TTTGGGTCGAGAACCACGAG
